# Special

**Published:** 1888-04

**Authors:** 


### HALL'S
### JOURNAL OF HEALTH

TRUTH DEMANDS NO SACRIFICE; ERROR CAN MAKE NONE.

VOL. 35. APRIL, 1888. No. 4.

### SPECIAL.

We have a limited supply of back numbers of HALL'S JOURNAL OF HEALTH for the years 1886 and 1887, which we offer to send to any address in the United States or Canada, post paid, as follows:

Sets of 1886, except January number,............25 cents.

Sets of 1887, Complete,.........................30 cents.

